# Regulation of Osteoblast Differentiation by Cytokine Networks

**DOI:** 10.3390/ijms22062851

**Published:** 2021-03-11

**Authors:** Dulshara Sachini Amarasekara, Sumi Kim, Jaerang Rho

**Affiliations:** 1 Department of Zoology and Environment Sciences, Faculty of Science, University of Colombo, Colombo 00300, Sri Lanka; sachini@zoology.cmb.ac.lk; 2 Department of Microbiology and Molecular Biology, Chungnam National University, Daejeon 34134, Korea; na12423@naver.com

**Keywords:** osteoblast, osteoblast differentiation, osteoblastogenesis, cytokine, signaling pathway

## Abstract

Osteoblasts, which are bone-forming cells, play pivotal roles in bone modeling and remodeling. Osteoblast differentiation, also known as osteoblastogenesis, is orchestrated by transcription factors, such as runt-related transcription factor 1/2, osterix, activating transcription factor 4, special AT-rich sequence-binding protein 2 and activator protein-1. Osteoblastogenesis is regulated by a network of cytokines under physiological and pathophysiological conditions. Osteoblastogenic cytokines, such as interleukin-10 (IL-10), IL-11, IL-18, interferon-γ (IFN-γ), cardiotrophin-1 and oncostatin M, promote osteoblastogenesis, whereas anti-osteoblastogenic cytokines, such as tumor necrosis factor-α (TNF-α), TNF-β, IL-1α, IL-4, IL-7, IL-12, IL-13, IL-23, IFN-α, IFN-β, leukemia inhibitory factor, cardiotrophin-like cytokine, and ciliary neurotrophic factor, downregulate osteoblastogenesis. Although there are gaps in the body of knowledge regarding the interplay of cytokine networks in osteoblastogenesis, cytokines appear to be potential therapeutic targets in bone-related diseases. Thus, in this study, we review and discuss our osteoblast, osteoblast differentiation, osteoblastogenesis, cytokines, signaling pathway of cytokine networks in osteoblastogenesis.

## 1. Background

Bone modeling initially occurs during development, where there are two modes of bone development: intramembranous ossification and endochondral ossification [1]. In intramembranous ossification, mesenchymal tissues are directly converted to bone, while in endochondral ossification, mesenchymal tissues are differentiated into cartilage before being replaced by bone [1]. Bone remodeling is a life-long process in which the volume of bone resorbed by osteoclasts (OCs) is restored by bone-forming osteoblasts (OBs) [2]. A balance between OC and OB activity is crucial in maintaining physiological bone turnover rates, and a flaw in this balance can lead to debilitating bone diseases, such as rheumatoid arthritis (RA), periodontal diseases, and osteoporosis [3]. Thus, maintaining the biomechanical integrity of bone by either modeling or remodeling is a complex process regulated by numerous cell lineages, transcription regulation, a network of cytokines, and growth factors [2,4]. Adequate understanding of the regulation of OC and OB activity in bone tissue is crucial for the development of novel therapeutics to manage bone-related diseases. We have previously reviewed the role played by cytokines in regulating OC differentiation, also known as osteoclastogenesis, under physiological and pathophysiological conditions [2,3]. In this article, we review the current knowledge of the impact of cytokines in OB differentiation, also known as osteoblastogenesis.

## 2. OB Differentiation and Function

**Figure 1:**
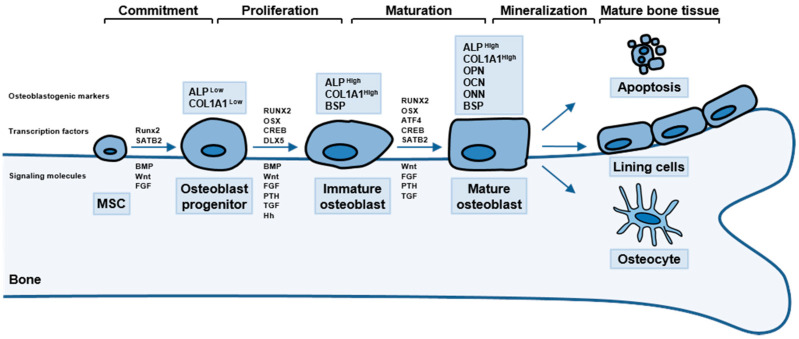
Schematic representation of osteoblast (OB) differentiation. MSC, mesenchymal stem cell. BMP, bone morphogenetic protein. FGF, fibroblast growth factor. RUNX2, runt-related transcription factor 2. PTH, parathyroid hormone. TGF, transforming growth factor. Hh, hedgehog. SATB2, special AT-rich sequence-binding protein 2. OSX, osterix. ATF4, activating transcription factor 4. CREB, cAMP-responsive element-binding. ALP, alkaline phosphatase. OPN, osteopontin. OCN, osteocalcin. ONN, osteonectin. BSP, bone sialoprotein. COL1A1, collagen type 1 alpha 1 chain.

OBs, which are bone-forming cells, are small mononucleated cells of mesenchymal stem cell (MSC) origin [4]. OBs are usually cuboid in shape but can be found in morphologically diverse round, flat and cylindrical forms [5]. The sequential action of cytokine networks and transcription factors results in the differentiation of OB lineage cells from mesenchymal precursors (Figure 1) [4].

OB lineage progenitor cells undergo three developmental stages: (1) cell proliferation, (2) extracellular matrix (ECM) secretion and matrix maturation and (3) matrix mineralization [6]. Following OB lineage commitment, pre-OBs undergo active proliferation and express collagen, fibronectin, osteopontin (OPN) and transforming growth factor-β (TGF-β) receptor 1 [7,8,9]. In the second stage, cell proliferation is downregulated, and immature OBs differentiate into mature OBs that secrete collagen type 1 alpha 1 chain (COL1A1) as the major constituent of the ECM and express alkaline phosphatase (ALP) to mature the ECM [7,8,9]. Upon completion of matrix maturation, matrix mineralization occurs in a highly ordered process via the expression of various osteoblastogenic markers, such as OPN, osteocalcin (OCN), and bone sialoprotein (BSP), with continued expression of ALP and COL1A1 [4,7]. OCN regulates calcium metabolism and promotes the deposition of minerals in the ECM, OPN promotes bone formation and mineralization, and BSP promotes mineralization regulating hydroxyapatite crystal formation [10,11]. Finally, mature OBs undergo apoptosis, become bone-lining cells or progressively incorporate into the bone matrix as terminally differentiated osteocytes (OSs) [6].

OBs orchestrate the bone remodeling process by regulating bone-resorbing OC differentiation and function through the production of two essential cytokines: receptor activator of nuclear factor-kappa B (RANK) ligand (RANKL) and macrophage colony-stimulating factor (M-CSF) [2]. The binding of RANKL and M-CSF to receptors RANK and c-fms, respectively, on the surface of OC progenitors, induces a number of downstream signaling cascades, ultimately activating nuclear factor of activated T cells c1, a master transcription factor of osteoclastogenesis, leading to enhanced OC differentiation, proliferation and survival [2]. Moreover, OBs secrete osteoprotegerin (OPG), a key negative regulator of osteoclastogenesis that binds with RANKL, thereby hindering RANKL-RANK interaction [2]. Therefore, OBs are vital for maintaining balance in bone homeostasis by regulating the RANK/RANKL/OPG axis [2,12].

## 3. Transcriptional Regulation in Osteoblastogenesis

Osteoblastogenesis is regulated by multiple cytokines and hormone signaling cascades, resulting in subsequent activation of downstream transcription factors [13,14]. Among the downstream transcription factors, runt-related related transcription factor 2 (RUNX2/CBFA1/AML3/PEBP2αA) acts as the master transcription factor leading to the expression of osteoblastogenic markers, such as ALP, OCN, OPN, osteonectin (ONN), BSP, and COL1A1, in osteoblastogenesis [15,16,17,18,19]. *Runx2*-deficient mice exhibit defects in endochondral and intramembranous bone formation [16,17,18]. In osteoblastogenesis, RUNX2 is marginally expressed in uncommitted MSCs and elevated throughout the proliferation of pre-OBs [20,21]. RUNX2 level peaks at the immature OB stage and decreases in the maturation stage [20,21]. RUNX2 enhances the expression of osterix (OSX/Sp7), an essential transcription factor for OB commitment and differentiation [22,23]. OSX can induce the expression of ALP, OCN, OPN, ONN, BSP, and COL1A1 [24]. *Osx*-deficient mice also exhibit defects in bone formation due to the complete loss of OBs [22,25]. However, RUNX2 expression remains unaltered in *Osx*-deficient mice, demonstrating that RUNX2 is upstream of OSX in osteoblastogenesis [22,25]. The number of OB progenitors and their proliferation was lower in the calvariae of *Runx2*-deficient mice, whereas *Osx*-deficient mice had OB progenitors in abundance with adequate proliferation, demonstrating that RUNX2 is required for the expansion of OB progenitors [25]. Consequently, the transcription factors RUNX2 and OSX are of high importance in osteoblastogenesis.

Activating transcription factor 4 (ATF4), belonging to the cAMP-responsive element-binding (CREB) protein family of transcription factors, is another crucial transcription factor in osteoblastogenesis [26,27]. ATF4 promotes osteoblastogenesis through direct interaction with RUNX2 to enhance OCN expression [28]. *ATF4*-deficient mice are reported to have severe osteoporosis, osteodysplasty, and altered bone mineralization with impaired terminal differentiation of OBs, probably due to reduced expression of COL1A1, OCN, and BSP [26]. Moreover, ATF4 can promote osteoblastogenesis indirectly by modulating β-catenin levels in MSCs [29]. Thus, ATF4 expression is limited in committed OB lineage cells, whereas RUNX2 and OSX are more broadly expressed from OB lineage commitment to maturation during osteoblastogenesis (Figure 1) [27]. Taken together, ATF4 is a direct or indirect transcriptional regulator of osteoblastogenic marker expression in osteoblastogenesis.

A recent study implicated RUNX1 in promoting endochondral ossification and osteoblastogenesis [30,31]. RUNX1 is expressed at all stages in OB lineage cells [32]. In a conditional knockout study of *Runx1^flox/flox^/Osx-Cre* mice, RUNX 1 deficiency resulted in decreased bone density by downregulating RUNX2, OSX, and ATF4 expression in OB lineage cells [30]. It was also determined that RUNX1 promotes RUNX2 and OCN expression by directly binding to the promoter regions of the *RUNX2* and *OCN* genes [30]. Furthermore, RUNX1 can improve OB lineage commitment and promote bone formation by upregulating the bone morphogenetic protein-7 (BMP-7) and Wnt/β-catenin pathways [32]. Therefore, RUNX1 has emerged as a novel regulator of osteoblastogenesis.

A crucial role of activator protein-1 (AP-1) and special AT-rich sequence-binding protein 2 (SATB2) in OB differentiation and function is also evident from previous studies [33,34,35]. AP-1 is a dimeric transcription factor that is primarily composed of c-jun, c-fos, and ATF family dimers [33]. AP-1 activation is induced by stimulation of osteoblastogenic factors, such as parathyroid hormone (PTH), TGF-β and vitamin D, in osteoblastogenesis [33,34,36]. Deficiency of c-fos and ATF members in mice has highlighted their importance in OB differentiation and function [33]. Other AP-1 members, such as Fra-1, Fra-2, and ΔFosB (FosB2), are implicated in promoting OB differentiation and function [37]. SATB2 is also implicated in promoting OB differentiation and bone regeneration by inducing RUNX2/ATF4-mediated expression of osteoblastogenic markers, such as OCN and BSP [35,38]. In addition, *SATB2*-deficient mice exhibit defects in OB differentiation and function, leading to delayed bone formation and mineralization [35]. A recent study also demonstrated that SATB2 promotes OB progenitor proliferation [39].

## 4. Signaling Pathways in Osteoblastogenesis

**Figure 2:**
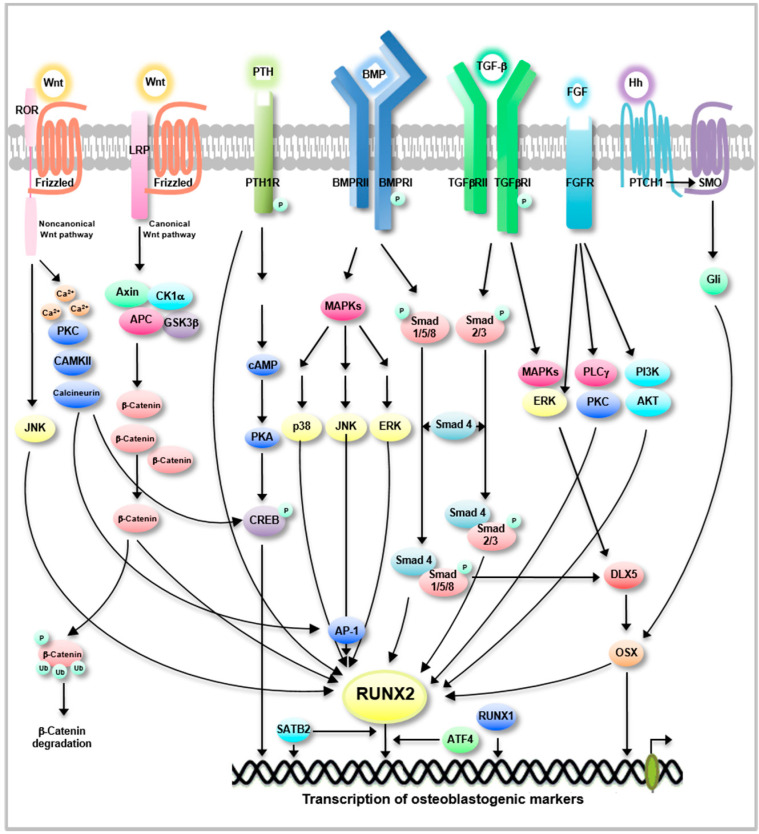
Key signaling pathways in osteoblastogenesis. BMP, bone morphogenetic protein. BMPR, BMP receptor. PTH, parathyroid hormone. PTH1R, PTH receptor 1. TGF-β, transforming growth factor-β. TGFβR, TGF-β receptor. FGF, fibroblast growth factor. FGFR, FGF receptor. Hh, hedgehog. PTCH1, patched 1. SMO, smoothened. RUNX1/2, runt-related transcription factor 1/2. OSX, osterix. ATF4, activating transcription factor 4. SATB2, special AT-rich sequence-binding protein 2. AP-1, activator protein-1. ROR, receptor tyrosine kinase-like orphan receptor. JNK, c-jun N-terminal kinase. CAMKII, calmodulin-dependent kinase II. PKC, protein kinase C. LRP, low-density lipoprotein receptor-related protein. APC, adenomatosis polyposis coli. CK1α, casein kinase 1α. GSK3β, glycogen synthase kinase 3β. cAMP, cyclic adenosine monophosphate. PKA, protein kinase A. CREB, cAMP-responsive element-binding. DLX5, distal-less homeobox 5. MAPK, mitogen-activated protein kinase. ERK, extracellular receptor kinase. PLC, phospholipase. AKT, protein kinase B. Gli, glioma-associated oncogene. Ub, Ubiquitin.

Osteoblastogenesis is regulated by multiple signaling pathways, including Wnt, PTH, BMP, TGF-β, fibroblast growth factor (FGF), and hedgehog (Hh) (Figure 2) [40]. The Wnt signaling pathway plays a pivotal role in promoting OB differentiation, proliferation, and maturation [40,41]. Wnt signaling can be categorized into two pathways: the canonical Wnt pathway and the non-canonical pathway [41]. The canonical Wnt pathway, also called the Wnt/β-catenin-dependent pathway, is best understood for its role in bone regeneration and repair [42]. In osteoblastogenesis, Wnt ligand binding to its receptors activates downstream signaling cascades, resulting in β-catenin translocation into the nucleus, thereby enhancing osteoblastogenic target gene expression (Figure 2) [4,43]. In the absence of Wnt ligand binding (or inactive state), β-catenin is phosphorylated by β-catenin destruction complex proteins, including axin, adenomatous polyposis coli, glycogen synthase kinase-3 β, and casein kinase-1 [43]. Phosphorylated β-catenin is ubiquitinated by β-TrCP ubiquitin E3 ligase and degraded by the ubiquitin-dependent proteasomal system (Figure 2) [43]. Non-canonical Wnt signaling induced by Wnt5a or Wnt11 binding to a receptor complex consisting of frizzled and the receptor tyrosine kinase-like orphan receptor (ROR) coreceptor transduces signals through c-jun N-terminal kinase (JNK) activation to induce RUNX2 (Figure 2) [44]. *Wnt5a*-deficient mice had low OB numbers and reduced bone mass, indicating that Wnt5a is important for MSC lineage commitment to OB differentiation [45,46]. The Wnt/calcium pathway, one of the non-canonical Wnt signaling pathways, increases intracellular calcium levels to activate calmodulin-dependent kinase II, protein kinase C (PKC), and calcineurin, leading to the induction of AP-1 transcription factors (Figure 2) [47].

BMPs belong to the TGF-β superfamily and are reported to be osteoblastogenic factors [48]. In particular, it has been well-documented that BMP-2 is a potent inducer of osteoblastogenesis by activating the Smad signaling pathway [49,50]. BMPs bind to serine/threonine kinase receptor II and activate receptor I to transmit signals through Smad1/5/8 (Figure 2) [49,50,51]. Smad1/5/8 complexed with Smad4 is translocated to the nucleus to activate RUNX2, leading to enhanced expression of osteoblastogenic markers [49,50,51]. Moreover, treatment with antagonistic antibodies against BMP-2, -4, and -7 can downregulate the expression of osteoblastogenic markers, such as ALP, OCN, and BSP, revealing that BMP-mediated signaling is crucial for RUNX2-mediated osteoblastogenesis [51]. BMP-2/Smad signaling is also reported to induce OSX through distal-less homeobox 5 (DLX5) induction in a RUNX2-independent manner [52]. In the cranial structure of the mesenchyme, DLX5 is reported to induce osteoblastogenesis by inducing RUNX2-mediated ALP and OPN expression, revealing that DLX5 is an upstream regulator of RUNX2 [53].

TGF-β can directly induce osteoblastogenesis from OB progenitor cells [54]. The ability of TGF-β to enhance OB proliferation, inhibit OB apoptosis, recruit OB precursors to the bone-forming site and produce ECM during osteoblastogenesis has been well-documented [55,56,57,58]. TGF-β binding to TGFβRI and TGFβRII triggers downstream signaling through Smad2/3 (Figure 2) [59]. Activated Smad2/3 forms a complex with Smad4 and undergoes nuclear translocation to induce RUNX2-mediated osteoblastogenic gene expression [59]. Moreover, TGF-β and BMPs can induce TGF-β activation kinase 1 to activate RUNX2 through the mitogen-activated protein kinase (MAPK) signaling pathway [59].

The FGF/FGF receptor (FGFR)-mediated signaling cascade regulates OB progenitor proliferation, maturation, and apoptosis [60]. FGF/FGFR signaling is reported to induce RUNX2 activation, leading to enhanced expression of osteoblastogenic markers, such as ALP, OCN, and COL1A1, through downstream signaling of phosphatidylinositol-3-kinase (PI3K)/protein kinase B (AKT), phospholipase γ/PKCα and extracellular receptor kinase (ERK)1/2 (Figure 2) [60]. *FGF2*-deficient mice exhibit decreased bone mass and bone formation, and *FGF18*-deficient mice show delayed ossification [61,62,63]. Moreover, *FGFR2*-deficient mice with conditional deletion of FGFR2 exhibit decreased bone formation and reduced proliferation of OB progenitor cells [64].

PTH is a positive regulator of osteoblastogenesis [65]. PTH induces the proliferation of OB progenitor cells, OB lineage commitment, and maturation in osteoblastogenesis [66]. PTH binding to the PTH receptor induces cAMP/PKA downstream signaling to phosphorylate and activate CREB, a member of a large family of basic leucine zipper domain DNA-binding proteins (Figure 2) [65,67,68]. Activated CREB induces the expression of osteoblastogenic markers, such as OCN and BSP, leading to bone formation [68,69]. Moreover, PTH-activated CREB effectively induces BMP-2 expression [68]. Mice with a conditional deletion of the G-protein coupled type 1 PTH receptor in OBs exhibit disrupted trabecular bone formation resulting from reduced OB activity [70].

Hh signaling is involved in promoting osteoblastogenesis [71,72]. Hh signaling activates glioma-associated oncogene (Gli) transcription factors by releasing the membrane protein smoothened, which triggers signaling cascade, and the activated Gli transcription factor travels to the nucleus to stimulate RUNX2/OSX activation in osteoblastogenesis (Figure 2) [73]. In rat MSCs, Hh also induces OB proliferation and differentiation by RUNX2-induced ALP, OCN, and COL1A1 expression [74]. Moreover, Hh and BMP synergistically induce osteoblastogenesis in the endochondral skeleton [75].

## 5. Cytokine Regulation of Osteoblastogenesis

Osteoblastogenesis is tightly regulated by complex cytokine networks under physiological and pathophysiological conditions [76]. Osteoblastogenic cytokines, such as interleukin-10 (IL-10), IL-11, IL-18, interferon-γ (IFN-γ), cardiotrophin-1 (CT-1), and oncostatin M (OSM), promote osteoblastogenesis, whereas anti-osteoblastogenic cytokines, such as tumor necrosis factor-α (TNF-α), TNF-β, IL-1α, IL-4, IL-7, IL-12, IL-13, IL-23, IFN-α, IFN-β, leukemia inhibitory factor (LIF), cardiotrophin-like cytokine (CLC) and ciliary neurotrophic factor (CNTF), downregulate osteoblastogenesis (Table 1).

TNF-α is a proinflammatory cytokine that plays an important role in bone diseases [3]. TNF-α inhibits RUNX2 expression and RUNX2-induced osteoblastogenic marker expression in OB precursors and MC3T3-E1 preosteoblastic cells [91,92]. TNF-α blocks osteoblastogenic marker expression by osteoblastogenic mediator β-glycerophosphate-induced RUNX2 activation via the TNF type 1 receptor [93]. TNF-α is also reported to inhibit BMP-induced osteoblastogenesis by activating JNK signaling and suppressing BMP/Smad signaling [94]. In addition, the expression of nephronectin, an extracellular matrix protein considered to be a positive regulator of osteoblastogenesis, is suppressed by TNF-α in MC3T3-E1 preosteoblastic cells [95]. TNF-α abrogates OB lineage commitment in osteoblastogenesis by increasing ubiquitin E3 ligase Wwp1 expression, leading to proteasomal degradation of the AP-1 transcription factor [96]. TNF-α also inhibits the expression of osteoblastogenic transcription factor SATB2 via the BMP/Smad, NF-κB and MAPK signaling pathways in osteoblastogenesis [97]. In estrogen deficiency-induced osteoporosis, TNF-α inhibits OB proliferation and differentiation by upregulating P2YR expression via the ERK/JNK signaling pathways [98]. In addition to TNF-α, a recent study reported that TNF-β inhibits the early stage of osteoblastogenic differentiation from MSCs by downregulating RUNX2 and activating NF-κB [99]. Taken together, TNF-α and TNF-β are known to be strong anti-osteoblastogenic cytokines.

IL-1 is a potent proinflammatory cytokine that exists in two forms: IL-1α and IL-1β [100]. IL-1α induces OB apoptosis and inhibits osteoblastogenesis by activating the JNK/p38 MAPK pathway, while IL-1β induces OB differentiation from MSCs and matrix mineralization through activation of the non-canonical Wnt (Wnt5a/ROR2) signaling pathway [100,119]. However, inhibitory functions of IL-1β in OB differentiation and bone formation were also reported [120]. Under inflammatory conditions, IL-1β and TNF-α are reported to suppress OB differentiation from MSCs and matrix mineralization by downregulating osteoblastogenic markers, such as RUNX2, OSX, ALP, and COL1A1 [121]. IL-18, a proinflammatory cytokine belonging to the IL-1 family, acts as a mitogen in OB proliferation [82]. IL-37, an anti-inflammatory cytokine belonging to the IL-1 family, promotes osteoblastogenesis by inducing osteoblastogenic markers, such as RUNX2, ALP, OCN, and COL1A1, by activating the PI3K/AKT pathway [141]. In contrast, the anti-osteoblastogenic role of IL-37, which suppresses BMP-2 and ALP expression, has been documented in chronic inflammatory conditions, such as calcific aortic valve disease [142]. Thus, IL-1 family cytokines play important roles in osteoblastogenesis, depending on physiological and pathophysiological status.

IL-3 is a multicolony stimulating factor produced by T cells [2,143]. In osteoblastogenesis, IL-3 induces OB differentiation and matrix mineralization by activating RUNX2 and OSX, leading to the expression of osteoblastogenic markers, such as ALP, OPN, OCN, and COL1A1 [122]. IL-3 indirectly induces osteoblastogenesis by inducing BMP-2 expression through the Janus kinase (JAK)/signal transducer and activator of transcription 3 (STAT) signaling pathway [122]. In contrast, it has been reported that BMP-2-mediated osteoblastogenesis is inhibited in multiple myeloma patients with high IL-3 levels [123]. Thus, IL-3 plays an important role in osteoblastogenesis depending on physiological and pathophysiological status.

IL-4 is an anti-inflammatory cytokine that shares some biological similarities with IL-13 [101]. IL-4 and IL-13 inhibit osteoblastogenesis by downregulating PTH-induced ALP activity in MC3T3-E1 preosteoblastic cells [101]. IL-4 and IL-13 also inhibit the proliferation of human OBs and induce IL-6 production in these cells to regulate OC recruitment [102,103]. IL-4 inhibits ALP expression and matrix mineralization in MC3T3 preosteoblastic cells [104]. Taken together, these findings indicate that IL-4 and IL-13 are anti-osteoblastogenic cytokines.

The IL-6 family of cytokines consists of IL-6, IL-11, OSM, CT-1, LIF, CLC, and CNTF [144]. In murine calvarial OBs and MC3T3-E1 preosteoblastic cells, IL-6 inhibits osteoblastogenesis and matrix mineralization by downregulating osteoblastogenic markers, such as RUNX2, OSX, and OCN [124]. Moreover, IL-6 depletion increases BMP2/7-induced osteoblastogenesis in KS483 preosteoblastic cells [125]. In contrast, in periodontal ligament cells, IL-6 exerts osteoblastogenic effects by enhancing RUNX2 and ALP expression [126]. IL-6 also increases ALP activity and matrix mineralization in human adipose stem cells [127]. Moreover, IL-6 accelerates matrix mineralization through STAT3-dependent ROR2 induction in human adipose tissue-derived MSCs [128]. Thus, the functional role of IL-6 in osteoblastogenesis is still controversial.

The IL-6 family member IL-11 is produced in response to IL-1, TNF-α, TGF-β, PTH, and mechanical stress in OB lineage cells [145,146,147]. IL-11 induces osteoblastogenesis by suppressing Dickkopf1/2 inhibitors of Wnt signaling [79]. Moreover, IL-11 induces osteoblastogenesis synergistically with BMP-2 by increasing osteoblastogenic markers, such as ALP, OCN, BSP and PTH receptor [80,81]. Similarly, OSM directly stimulates OB commitment from MSCs, OB differentiation, and matrix mineralization by suppressing sclerostin, a potent inhibitor of bone formation secreted by OSs [88]. CT-1 is capable of increasing OB activity through the activation of RUNX2, CAAT/enhancer-binding protein-δ (C/EBP-δ) and C/EBP-β [89,90]. Moreover, *CT-1*-deficient mice showed reduced OB numbers [89]. However, the IL-6 family member LIF inhibits osteoblastogenesis at the early stages through the STAT3 signaling pathway [110,111]. LIF receptor overexpression in human MSCs suppresses osteoblastogenesis by downregulating RUNX2, ALP, and ONN, while LIF receptor depletion by siRNA knockdown enhances osteoblastogenesis [112]. The IL-6 family member CLC1 is reported to prevent OB differentiation from MSCs and matrix mineralization through STAT1/3 signaling pathways [113]. CNTF, another member of the IL-6 family of cytokines, inhibits matrix mineralization and OSX expression in OBs [114]. *CNTF*-deficient mice exhibited increased OB numbers and high mineralization rates [114]. Moreover, myogenic CNTF suppresses the expression of osteoblastogenic markers, such as RUNX2, OSX, ALP, OCN, and PTH receptors, in murine calvarial OBs [115]. Taken together, in the IL-6 family members, IL-11, OSM, and CT-1 exert osteoblastogenic effects, while LIF, CLC, and CNTF are anti-osteoblastogenic cytokines.

IL-7 is a crucial cytokine in B and T cell lymphopoiesis [148]. It has been reported that direct injection of IL-7 in mice inhibits bone formation [105]. In periodontal ligament stem cells, IL-7 suppresses osteoblastogenesis by downregulating osteoblastogenic markers, such as RUNX2, OSX, ALP, and OCN, through the inactivation of the MAPK pathway [106]. In multiple myeloma, IL-7 is implicated in inhibiting bone formation by suppressing RUNX2 activity [107]. Moreover, in estrogen deficiency, IL-7 inhibits osteoblastogenesis by reducing RUNX2 activation [105]. Thus, IL-7 is a potent inhibitor of osteoblastogenesis in both physiological and pathophysiological states.

IL-10 is an anti-inflammatory cytokine [2]. The osteoblastogenic properties of IL-10 are less thoroughly documented than those of other cytokines. Low physiological concentrations of IL-10 induce osteoblastogenesis by activating the p38 MAPK signaling pathway in human MSCs, while higher pathological doses of IL-10 inhibit osteoblastogenesis by activating NF-κB signaling [77]. Moreover, *IL-10*-deficient mice exhibit reduced bone formation [78]. Thus, IL-10 can be considered a potential osteoblastogenic cytokine.

IL-12 and IL-23 are proinflammatory cytokines belonging to the IL-12 family [2,76,148]. *IL-12p40*-deficient mice, defective in both IL-12 and IL-23, have been reported to have enhanced bone formation in the distal femur [108]. Moreover, IL-12 and IL-23 indirectly inhibit osteoblastogenesis by stimulating CD4^+^ T cells [108]. Enhanced bone formation *IL-12p40*-deficient mice was protective against age-related bone loss [109]. Collectively, IL-12 and IL-23 are potential anti-osteoblastogenic cytokines.

IL-15 is a proinflammatory cytokine that shares most of its biological activities with IL-2 [149]. Elevated levels of IL-15 have been reported in inflammatory bone diseases, such as RA and periodontal disease [129]. IL-15 stimulates apoptosis of OBs via activation of NK cells [129]. However, IL-15Rα deficiency decreases OB function and bone mineralization [130]. Thus, the role of IL-15 in osteoblastogenesis remains controversial.

IL-17 is a proinflammatory cytokine predominantly produced by T helper 17 (Th17) cells, dendritic cells, and other immune cells [150]. It has been reported that OB maturation is stimulated by proinflammatory Th17 cells [131]. IL-17 produced by Th17 cells induces OB maturation of human MSCs [132]. Furthermore, IL-17 exhibits synergistic effects with BMP-2 in matrix mineralization and bone formation [132,133]. In a recent study, IL-17 was implicated in accelerating OB differentiation, matrix mineralization, and proliferation in mouse calvarial OBs [134]. Osteogenic differentiation of MSCs induced by IL-17 is further enhanced by coculture with OSs, indicating that IL-17 alters the MSC niche to induce osteoblastogenesis in cooperation with OSs [135]. In ankylosing spondylosis, IL-17 induces osteoblastogenesis by activating the JAK2/STAT3 pathway [136,137]. In contrast, IL-17 has been reported to inhibit osteoblastogenesis in rat calvarial OB precursors, with reduced expression of OSX, ALP, and OCN [138]. Similarly, an inhibitory effect of IL-17 on calvarial OB differentiation via regulation of canonical Wnt signaling pathway components has been reported in a spondyloarthritis model [139]. Furthermore, IL-17 inhibited BMP-2-induced osteoblastogenesis by downregulating osteoblastogenic markers, such as RUNX2, ALP, and OCN [140]. Thus, the role of IL-17 in osteoblastogenesis is still controversial.

IFN-γ is a well-known inhibitor of OC differentiation, but its role in osteoblastogenesis is also documented [2,151]. IFN-γ promotes osteoblastogenesis by inducing the expression of osteoblastogenic markers, such as RUNX2, OSX, ALP, and OCN [83,84,85]. IFN-γ deficiency or knockdown in human MSCs inhibits osteoblastogenesis by downregulating RUNX2 expression [86,87]. Moreover, IFN-γ receptor-deficient mice exhibit decreased OB differentiation capacity [87]. However, it has also been reported that IFN-γ and TNF-α synergistically promote OB apoptosis by inducing nitric oxide production or mitochondrial cytochrome c release, downregulating B cell lymphoma 2 expression and activating caspases [152,153]. IFN-α inhibits OB progenitor proliferation and differentiation by inhibiting ALP activity and downregulating BMP-2 expression [116]. Moreover, IFN-β exerts inhibitory effects on matrix mineralization by reducing the expression of COL1A1, fibronectin, fibulin, fibrillin, and laminin [117]. Moreover, it has been recently reported that DEF6, also known as IFN regulatory factor 4-binding protein, suppresses osteoblastogenesis via endogenous type 1 IFN-mediated feedback inhibition [118]. Taken together, these findings indicate that IFN-γ is an osteoblastogenic cytokine, although some exceptions may exist, while IFN-α/β is a potential anti-osteoblastogenic cytokine.

## 6. Concluding Remarks

Over the decades, the field of osteoimmunology has advanced to demonstrate the vital role played by cytokines in osteoblastogenesis and osteoclastogenesis and elucidate the potential use of such cytokines in clinical therapeutics. In particular, osteoblastogenic and anti-osteoblastogenic cytokines play an important role in osteoblastogenesis by linking the skeletal and immune systems. Dysregulation of osteoblastogenic and anti-osteoblastogenic cytokines can have a deleterious effect on bone metabolism homoeostasis. TNF-α, TNF-β, IL-1α, IL-4, IL-7, IL-12, IL-13, IL-23, IFN-α, IFN-β, LIF, CLC, and CNTF act as potent inhibitors of osteoblastogenesis, whereas IL-10, IL-11, IL-18, IFN-γ, CT-1, and OSM are osteoblastogenic (Table 1). Though each cytokine is supposed to have either stimulatory or inhibitory properties in osteoblastogenesis, the physiological mechanisms of action are complicated and possibly dependent on developmental stage, pathophysiological status, cytokine level, and the nature of the target cells.

## Figures and Tables

**Table 1:** Summary of the effects of osteoblastogenic and anti-osteoblastogenic factors in osteoblastogenesis.

Factor	Action	Ref.
**Osteoblastogenic factor**		
Wnt	induces RUNX2-mediated OB differentiation by canonical/non-canonical Wnt signaling	[41,42,43,44,45,46,47]
BMP	induces RUNX2/OSX-mediated OB differentiation by Smad dependent/independent signaling	[48,49,50,51,52,53]
TGF-β	induces RUNX2-mediated OB differentiation and inhibits OB apoptosis by Smad dependent/independent signaling	[54,55,56,57,58,59]
FGF	induces RUNX2-mediated OB differentiation/proliferation by PI3K/PLCγ/ERK signaling	[60,61,62,63,64]
PTH	induces CREB-mediated OB differentiation/proliferation by cAMP/PKA signaling	[65,66,67,68,69,70]
Hh	induces Gli/RUNX2/OSX-mediated OB differentiation/proliferation	[71,72,73,74,75]
IL-10	indirectly induces bone formation by p38 MAPK signaling	[77,78]
IL-11	induces OB differentiation by suppressing Wnt signaling inhibitor induces OB differentiation in synergy with BMP-2 signaling	[79,80,81]
IL-18	induces OB proliferation	[82]
IFN-γ	induces RUNX2/OSX-mediated OB differentiation	[83,84,85,86,87]
OSM	induces OB differentiation by suppressing bone formation inhibitor	[88]
CT-1	induces RUNX2-mediated OB differentiation	[89,90]
**Anti-osteoblastogenic factors**		
TNF-α	inhibits RUNX2- AP-1 or SATB2-mediated OB differentiation/proliferation	[91,92,93,94,95,96,97,98]
TNF-β	inhibits RUNX2-mediated OB differentiation	[99]
IL-1α	inhibits OB differentiation and induces OB apoptosis by JNK/p38 MAPK signaling	[100]
IL-4/13	inhibits PTH-induced OB differentiation/proliferation by downregulating PTH-mediated signaling	[101,102,103,104]
IL-7	inhibits RUNX2/OSX-mediated OB differentiation by downregulating MAPK signaling	[105,106,107]
IL-12/23	inhibits OB differentiation by stimulating CD4^+^ T cells	[108,109]
LIF	inhibits RUNX2-mediated OB differentiation by STAT3 signaling	[110,111,112]
CLC	inhibits OB differentiation by STAT1/3 signaling pathway	[113]
CNTF	inhibits RUNX2/OSX-mediated OB differentiation	[114,115]
IFN-α	inhibits BMP-induced OB differentiation/proliferation	[116]
IFN-β	inhibits bone formation and matrix mineralization	[117,118]
**Ambiguous roles**		
IL-1β	induces OB differentiation by non-canonical Wnt signaling	[119,120,121]
	inhibits RUNX2/OSX-mediated OB differentiation in inflammatory condition	
IL-3	induces RUNX2/OSX- or BMP-mediated OB differentiation	[122,123]
	inhibits BMP-induced OB differentiation in multiple myeloma	
IL-6	induces RUNX2-mediated OB differentiation and matrix mineralization by STAT3-dependent ROR2 induction	[124,125,126,127,128]
	inhibits RUNX2/OSX-mediated OB differentiation by downregulating BMP-mediated signaling	
IL-15	induces matrix mineralization	[129,130]
	induces OB apoptosis via NK cell activation	
IL-17	induces OB differentiation; exhibits synergistic effects with BMP signaling	[131,132,133,134,135,136,137,138,139,140]
	inhibits RUNX2/OSX-mediated or Wnt/BMP-induced OB differentiation	
IL-37	induces RUNX2-mediated OB differentiation by PI3K/AKT signaling	[141,142]
	inhibits BMP-induced OB differentiation in chronic inflammatory conditions	

Ref., references.

## Data Availability

Not applicable.
